# Large Nabothian Cyst: A Rare Cause of Nulliparous Prolapse

**DOI:** 10.1155/2012/192526

**Published:** 2012-06-13

**Authors:** Aruna Nigam, Deepti Choudhary, Chitra Raghunandan

**Affiliations:** ^1^Department of Obstetrics and Gynaecology, Lady Hardinge Medical College and SSK Hospital, Delhi 110001, India; ^2^Department of Obstetrics and Gynaecology, University College of Medical Sciences and Guru Teg Bahadur Hospital, University of Delhi, Dilshad Garden, Delhi 110095, India

## Abstract

Genital prolapse is commonly observed in postmenopausal and multiparous women, However, nulliparous women contribute to 2% of prevalence. We report a case of 21-year-old female who presented with a large nabothian cyst contributing to prolapse. This is the first case reported in the literature.

## 1. Introduction

Pelvic organ prolapse is an issue of major health concern. Genital prolapse is commonly observed in postmenopausal and multiparous women. However, nulliparous women contribute to 2% of prevalence [1]. The etiological factors implicated in the nulliparous include inherent defect in pelvic supports for example Ehler-Danlos syndrome, congenital shortness of vagina, and deep uterovesical and uterorectal pouches. It can also be attributed to spina bifida occulta and split pelvis which result in inherent weakness of pelvic floor support. Family history of prolapse also confines congenital nature of prolapse. We report a case of 21-year-old female who presented with a large nabothian cyst contributing to prolapse. This is the first case reported in the literature. 

## 2. Case Report

**Figure 1:**
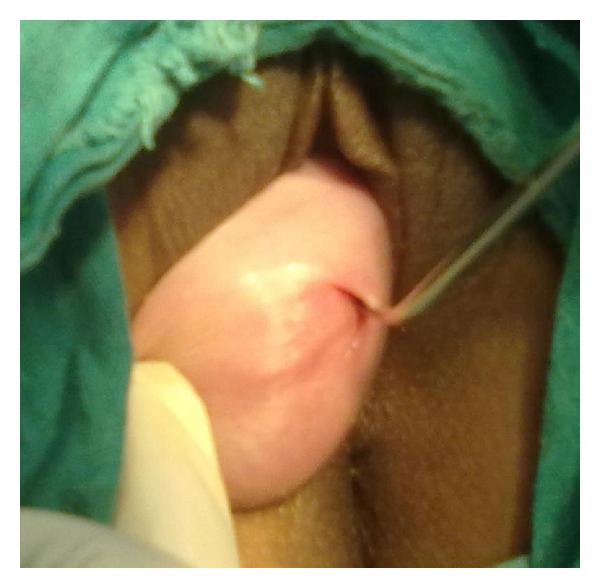
Third-degree cervical descent with a cyst inside on left side and external os (shown by a uterine sound).

A 21-years-old unmarried nulliparous female presented with complaints of something coming out of vagina for 8 years associated with discharge per vaginum. Menstruation was normal. There were no urinary or bowel complaints, chronic cough, constipation, heavy weight lifting, and no family history of similar problem. General physical and systemic examination including nervous system and vertebral column examination were unremarkable. Local genital examination revealed third-degree cervical descent manifesting as a cystic swelling protruding through the introitus. External os was compressed and deviated to one side due to swelling (Figure 1). Swelling was cystic and reducible. The pelvic examination revealed a normal-sized uterus and bilateral adnexa. Ultrasound showed a normal-sized uterus with normal myometrial echotexture and unremarkable endometrium with a 4.7 × 1.8 cm anechoic cyst in cervical region. 

**Figure 2:**
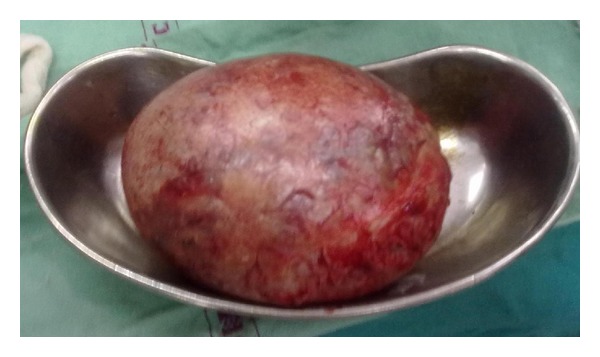
Completely removed Nabothian cyst.

Patient was planned for cystectomy and sling surgery. A large 4 × 5 cm cyst filled with white mucinous substance was removed from the cervix which was 35 grams in weight (Figure 2). After the cystectomy the cervical descent became second degree, so the sling surgery was postponed, and patient was asked to come for follow-up after 6 weeks, at which time, first-degree cervical descent was noted. Histopathology examination showed cyst wall lined with cuboidal epithelium suggestive of Nabothian cyst.

## 3. Discussion 

A nabothian cyst is a mucus-filled cyst on the surface of the cervix. They are most often caused when squamous epithelium blocks the opening of nabothian glands trapping mucosal secretion in small (2–10 mm diameter) subdermal pockets. They appear as firm bumps on the surface of cervix which may be single or in groups. Nabothian cysts are usually associated with chronic cervicitis, an inflammatory condition of cervix, and are harmless and usually disappear on their own. Nabothian cysts are not problematic unless they are sizeable and present secondary symptoms like in this case. 

Intraabdominal giant nabothian cyst has been reported [2]; however, the present case is the first being reported of a large nabothian cyst causing genital prolapse. Prolapse in this case appears to be due to the weight of the large nabothian cyst on the cervix which could have caused the elongation of the vaginal portion of the cervix.
